# Successful Consolidation/Maintenance Therapy with Single Agent Ibrutinib for Primary CNS Lymphoma after Initial Induction Therapy

**DOI:** 10.3390/neurolint14030046

**Published:** 2022-06-27

**Authors:** Steven Du, Daniela Bota, Xiao-Tang Kong

**Affiliations:** 1 Bachelor of Arts, University of Southern California, Los Angeles, CA 90089, USA; steven.du@pennmedicine.upenn.edu; 2 Department of Neurology, University of California Irvine, Irvine, CA 92697, USA; dbota@hs.uci.edu; 3 Department of Neurological Surgery, University of California Irvine, Irvine, CA 92697, USA

**Keywords:** primary central nervous system lymphoma, ibrutinib, consolidation therapy, maintenance therapy

## Abstract

Primary central nervous system lymphoma (PCNSL) is a rare and aggressive disease that originates from lymphocytes and develops in the central nervous system. There is no standard consolidation/maintenance therapy for PCNSL. While there exists a variety of options, the high chance of inferior outcomes for elderly patients and the risk of neurotoxicity requires exploration of alternative options for consolidation/maintenance therapy for PCNSL in the elderly population with CNS lymphoma. We treated one 77-year-old patient with single agent ibrutinib, a Bruton’s tyrosine kinase inhibitor that crosses the blood-brain-barrier, as consolidation/maintenance therapy after induction therapy with high-dose methotrexate (HD-MTX) and rituximab plus temozolomide. This treatment resulted in good tolerance, further resolution of a small residue lymphoma, and sustained remission. The patient has completed one year of consolidation/maintenance therapy and is currently under clinical and imaging surveillance. She has survived 27 months without recurrence since diagnosis. This case shows the potential effectiveness of single agent ibrutinib as consolidation/maintenance therapy for PCNSL after induction therapy. More cases are needed to confirm the findings.

## 1. Introduction

Primary central nervous system lymphoma (PCNSL) is a rare and aggressive sub-type of Non-Hodgkin’s Lymphoma. It has an incidence rate of 0.5 per 100,000 persons per year, mostly in people over 65 years old, and there are 1500 new cases diagnosed every year in the United States alone [1]. PCNSL originates from lymphocytes and develops in the central nervous system, usually in the brain, leptomeninges, eyes, or spinal cord. If left untreated, the median overall survival (OS) ranges from weeks to months [2]. Symptoms of PCNSL can progress rapidly and can include personality changes, cognitive impairments, focal neurological deficits, seizures, and increased intracranial pressure [3]. Contrast-enhanced cranial magnetic resonance imaging (MRI) is often used to image PCNSL, and biopsies are the gold standard test for its diagnosis [4].

Induction treatment for PCNSL mainly includes a single-agent or high-dose methotrexate-based (HD-MTX-based) chemotherapy regimen. The introduction of this type of treatment has improved the prognosis of PCNSL in recent decades, achieving complete or partial response in most PCNSL patients [5] after initial induction. Depending on physician preference and geographic location, a variety of these HD-MTX-based induction therapy regimens exist, such as single-agent HD-MTX, R-MVP, R-MT, MATRix, or R-MVBP [5]. Because of improvements in PCNSL treatment, the median overall survival of PCNSL has improved from 12.5 months to 26 months [6].

However, there is no standard consolidation therapy or maintenance therapy for PCNSL after induction therapy. Several consolidation/maintenance options exist, such as high-dose chemotherapy followed by autologous stem-cell transplant (HDC/ASCT), whole-brain radiotherapy (WBRT), traditional chemotherapy (cytarabine or etoposide with cytarabine), continuing monthly HD-MTX for up to a year, or observation (for patients who cannot tolerate additional treatment) [5,7], but there is a lack of strong evidence supporting the above consolidation/maintenance options as standard treatment due to risk of side effects and complications.

For example, in a phase III randomized trial with 318 patients enrolled, it was found that although adding WBRT consolidation therapy after HD-MTX-based chemotherapy did improve progression-free survival (PFS) from 12 months to 18 months compared to HD-MTX-based therapy alone, doing so did not improve OS; the median OS was 32.4 months in the group that underwent the additional WBRT and 37.1 months in the group that did not undergo WBRT [8]. Most notably, with WBRT, there was evidence of neurotoxicity in sustained complete remission survivors; clinically defined neurotoxicity was found in 71% of patients receiving WBRT and 46% of patients not receiving WBRT [8].

HDC/ASCT does have strong consolidation potential in younger patients. One study with 73 stem-cell transplanted patients showed that a median OS was not reached by follow-up after 57 months [9]. Another clinical trial followed a group of 26 transplanted patients for 45 months, ultimately yielding a two-year PFS and OS as high as 81%. However, there was a high mortality of 5–10% due to chemotoxicity occurring before and after transplant. In addition, elderly patients are often not eligible for HDC/ASCT due to risk of severe chemotoxicity [10]. Therefore, the use of HDC/ASCT as consolidation therapy is still controversial in various facilities.

Ibrutinib, a Bruton’s tyrosine kinase (BTK) inhibitor, is an FDA-approved drug for the treatment of mantle cell lymphoma, chronic lymphocytic leukemia, and marginal zone lymphoma [11,12]. Ibrutinib works by interfering with B-cell receptor (BCR) signaling, which effectively hinders the survival of lymphoma cells [13]. Since ibrutinib can cross the blood-brain-barrier, it has been used in studies involving the treatment of recurrent and refractory (r/r) CNS lymphoma, demonstrating good tolerance and survival benefit in patients with PCNSL [14,15].

As a result of the lack of consistent reliable consolidation/maintenance therapy for PCNSL for elderly patients, and because of the confirmed efficacy of ibrutinib as a single agent for the treatment of r/r PCNSL, we treated one elderly patient by using ibrutinib as consolidation/maintenance therapy after induction with HD-MTX and rituximab plus temozolomide, as she could not tolerate other options such as WBRT or HDC/ASCT. Ibrutinib treatment yielded good tolerance, further resolution of small residue lymphoma lesion, and sustained remission.

## 2. Case Report

**Figure 1:**
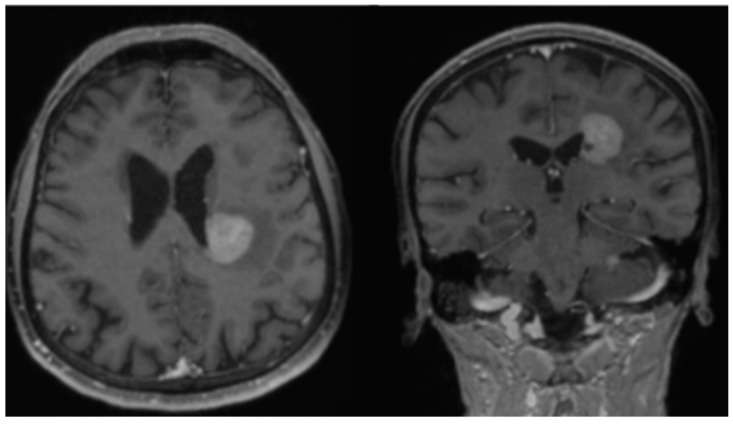
Brain MRI in January 2020, showing an enhancing mass on post-contrast T1 images.

**Figure 2:**
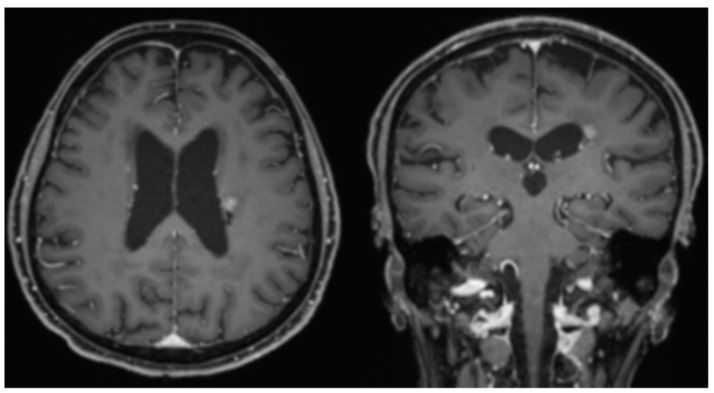
Brain MRI after 6 cycles of reduced-dose HD-MTX induction therapy, showing significant response of lymphoma to the treatment on post-contrast T1 images.

The patient is a 77-year-old female who initially presented with difficulty word-finding and writing at the end of 2019. She gradually developed right-sided weakness, aphasia, and progressive dysarthria, and was found to have multifocal lesions on brain MRI (Figure 1) in January 2020. The patient underwent biopsy of the largest lesion near the left lateral ventricle, which revealed diffuse large B-cell lymphoma. In accordance with NCCN guidelines, the patient began standard HD-MTX induction therapy [7]. She was started on HD-MTX at 6 g/m^2^ for the first cycle instead of 8 g/m^2^ due to reduced 24-h urine creatinine clearance (75 mL/min) [16]. She continued HD-MTX every two weeks but received a progressively reduced dosage of HD-MTX each cycle due to worsening renal function. Her brain MRI showed significant response after 6 cycles of reduced dosage of HD-MTX except small residue enhancement in the biopsy area at the left lateral ventricle aspect (Figure 2). After 6 cycles, her 24-h urine creatinine clearance dropped to 29 mL/min, so HD-MTX treatment was ultimately discontinued. Due to the patient’s inability to complete 8 cycles of HD-MTX as planned induction therapy, second-line treatment regimen rituximab and temozolomide [17] was initiated as continued induction therapy. The patient completed 8 doses of weekly rituximab and 8 weeks of one-week-on, one-week-off temozolomide [17]. The patient reached unconfirmed complete response (uCR) based on MRI imaging. For this patient, adjuvant temozolomide (daily for 5 days per 28-day cycle) as consolidation/maintenance therapy was initiated but ultimately discontinued due to renal intolerance.

**Figure 3:**
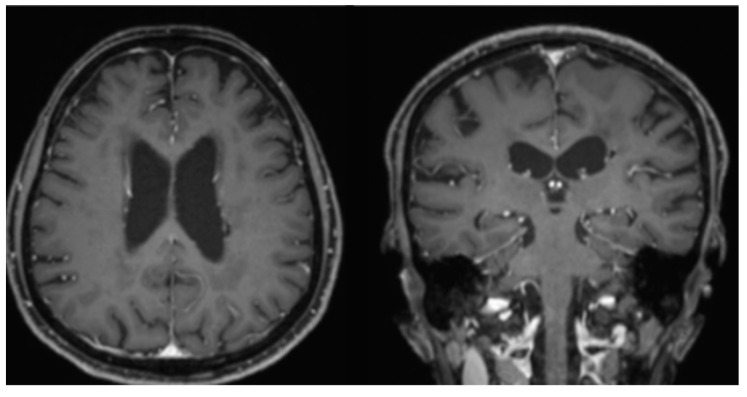
Brain MRI after 6 cycles of ibrutinib, showing resolution of small, enhanced lesion on post-contrast T1 images.

Ibrutinib as consolidation/maintenance therapy was started after discussing with the patient. The patient first underwent 6 cycles of 560 mg ibrutinib daily for 28 days per cycle, which resulted in resolution of the small lymphoma residue, and reached complete remission (Figure 3). However, due to neutropenia, which was a side effect of the ibrutinib, she began to receive a reduced dose of 420 mg daily for cycles 7 through 12. Regardless, the patient successfully completed one year of ibrutinib consolidation/maintenance therapy and underwent clinical and image surveillance over an additional 6 months. As of now, the patient has already survived 27 months since diagnosis without recurrence.

## 3. Results and Discussion

Our case highlights the potential effect of single agent ibrutinib as consolidation/maintenance therapy for PCNSL after HD-MTX and rituximab plus temozolomide induction therapy.

The standard induction treatment for PCNSL includes HD-MTX, which has been shown to achieve complete or partial response in most patients with PCNSL [5]; however, recurrence is frequent after 12 months. In our case, HD-MTX induction treatment yielded a good response after six out of eight cycles (Figure 2). HD-MTX was discontinued and replaced with second-line induction treatment (rituximab and temozolomide) due to the patient’s worsening renal function and decreased creatinine clearance.

Instead of using the combined three chemotherapy agents R-MT (rituximab at 375 mg/m^2^, MTX at 3.5 g/m^2^, and temozolomide) regimen, we chose single agent HD-MTX at 8 g/m^2^ for 8 cycles as initial induction therapy in our daily practice [16]. If complete remission is not achieved due to the interruption of 8 cycles of HD-MTX for a variety of causes, the patient will receive second line induction therapy with 8 doses of weekly rituximab (750 mg/m^2^) and 8 weeks of one-week-on and one-week-off temozolomide [17] to continue the induction. Then, 12 cycles of adjuvant temozolomide therapy (taken for 5 days over 28 days per cycle) will be used as consolidation/maintenance therapy [17].

There is currently no standard consolidation/maintenance therapy for PCNSL. The common options used for consolidation/maintenance treatment for PCNSL include HDC/ASCT, WBRT, traditional chemotherapy (cytarabine or etoposide with cytarabine), continued monthly HD-MTX for up to twelve cycles, or observation [5,7]. We did not use WBRT for our patient due to concern of neurocognitive impairments caused by WBRT [8]. The patient was also ineligible for HDC/ASCT due to high risk of complications for her age (77 years old). She also could not continue monthly HD-MTX or other chemotherapy agents because of worsening renal function. After discussing with the patient, she selected single agent ibrutinib as consolidation/maintenance treatment for her PCNSL.

Ibrutinib, a Bruton’s tyrosine kinase (BTK) inhibitor, works by interfering with B-cell receptor (BCR) signaling, which effectively hinders the survival of lymphoma cells [13]. Clinical study found that ibrutinib was able to cross the blood-brain-barrier and was detectable in the brain, CSF, and intraocular compartments; as a result, it was studied as a candidate for the treatment of PCNSL [14]. In a phase 1b study [18], ibrutinib was found to be safe and tolerable when used in combination with HD-MTX and rituximab as induction therapy regimen for r/r PCNSL patients; this treatment yielded a response rate of 80% (12 out of 15 patients). The PFS was 9.2 months, and the OS was not reached. In another earlier phase II study with 29 r/r PCNSL and 15 secondary CNS lymphoma (SCNSL) patients, treatment with single agent ibrutinib yielded an overall response rate of 78%. The median PFS was 4 months, and the median OS was 19.5 months [15].

Our patient has tolerated ibrutinib well, despite some neutropenia, and has completed ibrutinib as consolidation/maintenance therapy for 12 cycles (28 days per cycle) with no evidence of disease progression, showing potential effectiveness of ibrutinib as consolidation/maintenance therapy for PCNSL. Furthermore, the effect has been sustained after completion of the ibrutinib therapy, resulting in 27 months progression free survival as of writing this case report. We did not have a clear answer as to how long we should use ibrutinib as consolidation/maintenance therapy; based on the consolidation/maintenance chemotherapy regimen in literature and our experience, we believe one to two years of consolidation/maintenance therapy may be an appropriate length to achieve sustained remission.

Although ibrutinib has shown some promising outcomes for the treatment of PCNSL, it has not resulted in a complete response to every case, and the long-term remission is a challenge in most cases [14,15,18]. A second-generation irreversible oral BTK inhibitor, tirabrutinib, was developed by Ono Pharmaceutical in Japan [19]. In contrast to ibrutinib, which potently inhibits multiple off-target kinases, tirabrutinib is a highly selective BTK inhibitor, which may lead to less toxicities and better efficacy. A phase I/II study of tirabrutinib in the treatment of r/r PCNSL was completed by Narita et al. In this study, 44 patients with r/r PCNSL were enrolled. The overall response rate was 64%; PFS was 2.9 months and median OS was not reached. A best ORR was observed in the 480 mg dose group without fasting condition (100% CR/uCR in 4/4 cases with PFS of 11.1 months). This new generation BTK inhibitor was tolerated well except for one case of grade 5 adverse effect of pneumocystis jiroveci pneumonia and interstitial lung disease. Currently, an open-label phase II trial to investigate the efficacy and safety of tirabrutinib in patients with PCNSL is enrolling patients (ClinicalTrials.gov: NCT04947319). The outcome will be followed.

In addition to looking for newer generations of BTK inhibitors, studies have investigated potential biomarkers that can predict which patients will respond optimally to ibrutinib therapy. So far, there are no clear biomarkers that can predict which types of PCNSL patients are most likely to have a good response to ibrutinib treatment. Two potential biomarkers have been investigated. (1) Driver mutations behind PCNSL were studied by Bruno’s group [20]. Thirty-seven different gene mutations were associated with pathophysiology of PCNSL. Of them, driver mutations in the BCR pathway, including CD79BY196, which codes for pro-survival transcription factor nuclear factor -κB (NF-κB), were expected to be biomarkers for predicting the response of PCNSL to ibrutinib therapy. The rationale was that activated NF-κB, mediated by BTK, promotes PCNSL survival. Ibrutinib, a BTK inhibitor, should suppress the proliferation of CNS lymphoma with mutations in the BCR signaling pathway [21]. However, Grommes et al found that complete responses were achieved by ibrutinib therapy even in tumors without the driver mutations in the BCR signaling pathway. In addition, responses to ibrutinib therapy were observed in the patients who had tumors with mutations that were expected to be resistant to ibrutinib therapy [18]; therefore, the molecular basis of PCNSL has not become an accurate biomarker for predicting the response of PCNSL to ibrutinib therapy at this point. (2) Diffuse large B cell lymphoma (DLBCL), the vast major type of PCNSL, mainly includes two subtypes: activated B cell-like (ABC) and germinal center B cell-like (GCB), based on their gene expression profiles related to their potential cell of origin [22]. Of systemic DLBCL, the ABC subtype has had higher response rates than the GCB subtype to ibrutinib therapy (37% vs. 5%) [22]. However, the majority of CNS PCNSL are composed of ABC subtype DLBCL [23] and the responses to ibrutinib therapy were observed in both ABC and GCB subtypes [18]. Therefore, GCB subtype DLBCL was not excluded for being treated by ibrutinib [18]. Future studies to identify good responses to BTK inhibitors are needed.

## 4. Conclusions

Our case study showed that single agent ibrutinib may be a potential treatment option as consolidation/maintenance therapy for PCNSL following HD-MTX-based induction therapy, especially for patients whose age or comorbidities render them particularly susceptible to chemotoxicity from HDC/ASCT or prolonged consolidation/maintenance chemotherapy. Further study or clinical trial for the confirmation of our finding and investigation of the subtype that is responsive to ibrutinib therapy is warranted.

## Data Availability

Not applicable.
